# A correlative imaging based methodology for accurate quantitative assessment of bone formation in additive manufactured implants

**DOI:** 10.1007/s10856-016-5721-6

**Published:** 2016-05-06

**Authors:** Hua Geng, Naomi M. Todd, Aine Devlin-Mullin, Gowsihan Poologasundarampillai, Taek Bo Kim, Kamel Madi, Sarah Cartmell, Christopher A. Mitchell, Julian R. Jones, Peter D. Lee

**Affiliations:** School of Materials, The University of Manchester, Oxford Road, Manchester, M13 9PL UK; School of Biomedical Sciences, Centre for Molecular Biosciences (CMB), Ulster University, Coleraine, Northern Ireland, UK; Department of Materials, Imperial College London, London, SW7 2AZ UK

## Abstract

A correlative imaging methodology was developed to accurately quantify bone formation in the complex lattice structure of additive manufactured implants. Micro computed tomography (μCT) and histomorphometry were combined, integrating the best features from both, while demonstrating the limitations of each imaging modality. This semi-automatic methodology registered each modality using a coarse graining technique to speed the registration of 2D histology sections to high resolution 3D μCT datasets. Once registered, histomorphometric qualitative and quantitative bone descriptors were directly correlated to 3D quantitative bone descriptors, such as bone ingrowth and bone contact. The correlative imaging allowed the significant volumetric shrinkage of histology sections to be quantified for the first time (~15 %). This technique demonstrated the importance of location of the histological section, demonstrating that up to a 30 % offset can be introduced. The results were used to quantitatively demonstrate the effectiveness of 3D printed titanium lattice implants.

**Electronic supplementary material:**

The online version of this article (doi:10.1007/s10856-016-5721-6) contains supplementary material, which is available to authorized users.

## Introduction

Histology remains the gold standard for assessing bone formation within implants because it provides information with a high level of specificity in terms of cellular makeup coupled with excellent image resolution. Additionally, quantitative results, such as area-to-volume ratio, perimeter-to-area ratio [1] and trabecular width (Tb Wi) [2], can be acquired through histomorphometric analysis. Though histology can provide detailed and effective results, it has several drawbacks. Firstly, the preparation of bony sections containing metal implants is difficult and time-consuming [3] and requires special equipment [4]. Secondly, significant deformation and shrinkage of the sample may occur during processing [5], both altering the result and making it difficult to carry out further experiments. Thirdly, a single 2D image from a large volume sample may not represent bone formation over the entire implant. For a typical defect (3–5 mm diameter) in a small animal model, histology only looks at 3–5 % of the tissue when a 100 μm thickness bone implant section is prepared. Although serial sectioning may be an alternative, current methods are inadequate for serial sectioning of tissue in a metal implant without significant tissue loss. Additionally, histomorphometric evaluation of an image poses other difficulties, such as multiple tissue types and variations in staining uniformity within histological images making it difficult to form an objective judgment of overall regeneration in bone lesions [6].

More recently, μCT has been employed to image and characterise the three-dimensional (3D) structure of porous scaffolds [7, 8] and hard tissue [9, 10] in a non-invasive manner. Compared with histology, the substantial advantages of μCT lie in its simple sample preparation, ability to image whole 3D structures and high contrast (in the case of mineralised tissue) [11, 12]. Thus, it is an attractive technique to visualise and quantify bone regeneration within the defect site as well as around the implant [13, 14]. Additional information such as volume fraction on both the implant [7, 15] and newly formed bone has been derived.

Correlative imaging is the combination of multiple techniques, such as μCT with histology to provide complementary information [16] and it is a frequently used technique in medical imaging; for example, combining MRI with PET [17]. For bone formation, few authors have used μCT with histology [18–21] at the resolution required to achieve direct comparison of bone formation. Bernhardt et al. [18] were among the first to quantitatively analyse bone formation by using μCT and histology, but the implant had a simple geometric structure and the newly formed bone was quantified in predefined gaps only. Stalder et al. [22] reported the regenerative capacity of ceramic bone grafting materials with the dual application of μCT and histology. Their use of synchrotron tomography to image ceramic bone graft yields a much cleaner 3D dataset which is free of artefacts such as beam hardening and shadowing when bone is present in its vicinity [18, 22]. Synchrotron sources are, at present, scarce and do not completely eliminate the shadowing artefacts when titanium-implants are imaged. Therefore, there is a need for a new methodology to correlatively image and quantify bone formation in titanium-implants using histomorphometry and laboratory x-ray sources.

A direct comparison can be obtained simply by repeating a μCT scan of the histological sections [19], but it is limited to non-decalcified specimens. From the above, a robust technique that enables a direct comparison, taking advantage of the strengths of both laboratory μCT and histology is highly desirable. In order to match the histological section with its corresponding μCT region, a 2D–3D multimodal (different intensity level) registration is required.

2D–3D registration has been adopted in clinical applications such as image-guided interventions [23]. However, the specific problem of registering histology images to μCT registration has gained less attention and has never been validated, which may be due to the challenges associated with this process. Biological samples are subject to complex morphological deformations, staining artefacts, and missing tissue in individual preparation processing [5]. To address this issue, Museyko et al. [24] applied affine and elastic registration to tibiae and vertebra to estimate the effect of deformation. A main finding of Museyko’s study was that segmentation-based registration could achieve comparable accuracy as intensity-based registration.

Further, the issue of tissue shrinkage during histology section preparation is an important one when considering morphometry in fields such as biomechanics and implant surgery. However, to our knowledge, only one study [25] has attempted to quantify the volumetric shrinkage following histology procedures. This may be because of the difficulty in identifying the correspondence between different images.

Here, we report the first application of correlative imaging to quantify bone formation into a novel additive manufactured porous titanium scaffold. A 2D 50–80 μm thick haematoxylin and multiple staining solution stained histological section was registered into a 3D μCT dataset of implanted rat tibia from which the section was prepared. The presence of the non-deformed titanium scaffold allowed rigid registration of μCT and histology sections independently of bone and surrounding tissue. The quality and reliability of the registration was assessed to demonstrate a well-defined correlation between μCT and histology features. The rigid transformation was then used, for the first time, to achieve a comparable study of the bone ingrowth (BI) into porous titanium implant between μCT and histology. After that, a non-rigid registration was performed between the cortical bone and the histological image and its corresponding 2D μCT to quantify the volumetric shrinkage of bone in the preparation of the histological section.

## Materials and methods

### Production of the titanium scaffolds

The porous titanium scaffolds [26] were designed as a cylinder (diameter Ø = 3 mm, height h = 1.8 mm) consisting of orthogonal struts (Ø = 180 µm) and an overall porosity of 65 % [15].

The structures were fabricated from grade 1 commercially pure titanium (Sumitomo, Japan) using a selective laser melting technique [26]. The laser and scanner were computer-controlled using the Stereo Lithography (STL) data converted from the CAD data. The titanium powder was produced by gas-atomisation with a median particle size of 28.5 μm. All implants were sterilised by immersion in 200 μl of 70 % ethanol for 2 h. The ethanol was removed, then the samples were further sterilised with dry heat (200 °C for 2 h) before implantation.

### Animal experiment

A total number of 12 male Wistar rats (12 weeks old; body mass 250–300 g) were used in this study. Animal protocols were conducted in accordance with the institutional (Ulster University, United Kingdom (UK)) and national guidelines for animal care and welfare. All rats were anaesthetised by isoflourane gas inhalation followed by an intraperitoneal injection of 0.5 ml/100 g bodyweight of a mixture of 2 ml Ketaset (100 mg/ml), 1 ml Xylapan (20 mg/ml) and 5 ml phosphate buffered saline (PBS, pH 7.4). A deep plane of anaesthesia was confirmed by the loss of pedal reflex. The animals were premedicated with 1.5 ml of subcutaneous injection of Metacam (Boehringer Ingelheim, Germany) prepared in water (1:10 dilution) to reduce postoperative pain. The lower right leg was shaved with mechanical hair clippers. The area was cleared of all hair using depilatory cream (Veet^®^, Rickitt Benckiser Group Plc, UK), cleaned and sterilized using three consecutive washes of pre-warmed chlorhexidin (Hibisrub^®^, Regent Medical Ltd, UK), followed by 70 % isopropanol. Throughout the duration of the surgical procedure, animals were kept warm on a heating mat (37 °C). A 15 mm full-thickness longitudinal skin incision was made above the middle third of the medial aspect of the tibia. The skin flap was opened using fine spreaders (InterFocus Ltd, Cambridge, UK) exposing the underlying tibia. The tibial surface was cleared of connective tissue and periosteum. A 3 mm circular defect was created using a trephine burr, which extended into the level of the marrow cavity. The defect site was continuously cleared of blood and bone fragments with the aid of suction and the tissue kept moist by saline irrigation. During the surgical procedure, a suture was used to stabilise the porous titanium scaffold. After insertion of the scaffold, the overlying muscle was closed using with three sutures using 4-0 Ethicon Ethilon polyamide (Johnson & Johnson, U.S), one directly over the implant and one above and below the defect area. The skin was closed using a running suture. Post-operation, the animals received topical application of 0.2 % chloramphenicol solution to the sutured skin to prevent infection of the operative site followed by an intra-peritoneal administration of 5 ml of 5 % dextrose-saline solution. The rats were housed separately with 12 h light/dark cycles and were given unrestricted access to food and water. The right tibiae were harvested after either 2, 3, 4 or 6 weeks’ implantation (n = 3) for further evaluation.

### μCT image acquisition

The specimens were placed in an ABS plastic sample holder and scanned in a laboratory source μCT machine (nano-focus, Phoenix|x-ray General Electric Company, Measurement and Control, Wunstorf, Germany) with an isotropic voxel size of 9 μm (spatial resolution 19–24 μm). The scanning parameters were set to 85 kV and 111 μA. A 0.5 mm copper filter was placed in the x-ray path to reduce beam hardening. Each scan consisted of 1000 projections over 360°, with the sample rotated in equiangular steps along its longitudinal axis. For each projection, the exposure time was set to 2000 ms. Ring artefacts were reduced using detector jitter [27].

The projections were imported into the reconstruction software (Phoenix dato s| × 2 reconstruction) to generate 3D images of dimensions 990 × 990 × 1000 voxels. All specimens were scanned and reconstructed with identical settings.

### μCT image processing

After reconstruction, scan datasets were normalised to a predefined histogram. To remove digital noise and artefacts, the μCT volume was smoothed with anisotropic diffusion and edge-preserving filters, as described by Chao et al. [28]. The filtered volumes were then segmented into bone tissue and implant using an in-house algorithm. The implant was a high-signal low-noise region and was segmented by global thresholding. However, the bone region was more difficult to distinguish due to metal artefacts. An iterative resampling algorithm correction was applied to compensate for the effect of metal artefacts.

For all scans, the segmented bone and titanium phases were then registered with an average leg model and a cylindrical mask (whose dimensions were identical to those of the implant), respectively. 3D morphometric parameters were calculated in the volume of interest (VOI), which was defined by the overlap of the average leg model and the cylindrical mask. Bone ingrowth and bone contact (BC) were then measured for the new bone.

### Histology

Following fixation in 10 % neutral buffered formalin (Sigma-Aldrich, UK), all bone implant specimens were decalcified in 14 % EDTA (Sigma-Aldrich, UK), dehydrated through a series of increasing concentrations of ethanol, and embedded in LR white resin (TAAB Laboratories Equipment Ltd, UK) for sectioning.

The tissue blocks were then trimmed and a 100–200 μm section was cut using an EXAKT 310 Macro Band System with a diamond blade (EXAKT, USA), ground on an EXAKT 400CS grinding system (EXAKT, USA), using K800 and K1200 Grinding paper and P2500 and P4000 polishing paper (EXAKT, USA) down to a thickness of 50–80 μm. One transverse section was obtained from each specimen. All sections from the tibia were transverse, and the chosen section was at the location of the first cut exposing the implant. This meant the histological section was offset towards the implant edge.

All sections were stained with Gill’s Haematoxylin III (Fisher Scientific, Loughborough, UK) and multiple staining solution according to the following protocol. Briefly, slides with adhered sects. (50–80 μm) were placed in successive solutions of 1 % formic acid (2 min), 50 % ethanol (5 min), de-ionized water (5 min) followed by dropwise addition of Gill’s Haematoxylin to the slide for 30 min. Slides were subsequently rinsed well in water, stained with multiple staining solution (Polysciences Inc., Warrington, USA) for 20 min, further rinsed in distilled water, air dried and directly imaged via light microscopy using an Axio Scope 1 (Carl Zeiss, Germany) microscope at a range of objective magnifications (a magnification of 1.25 for a 5.23 μm/pixel and a magnification of 5 for a 1.26 μm/pixel).

### Registration

**Fig. 1:**
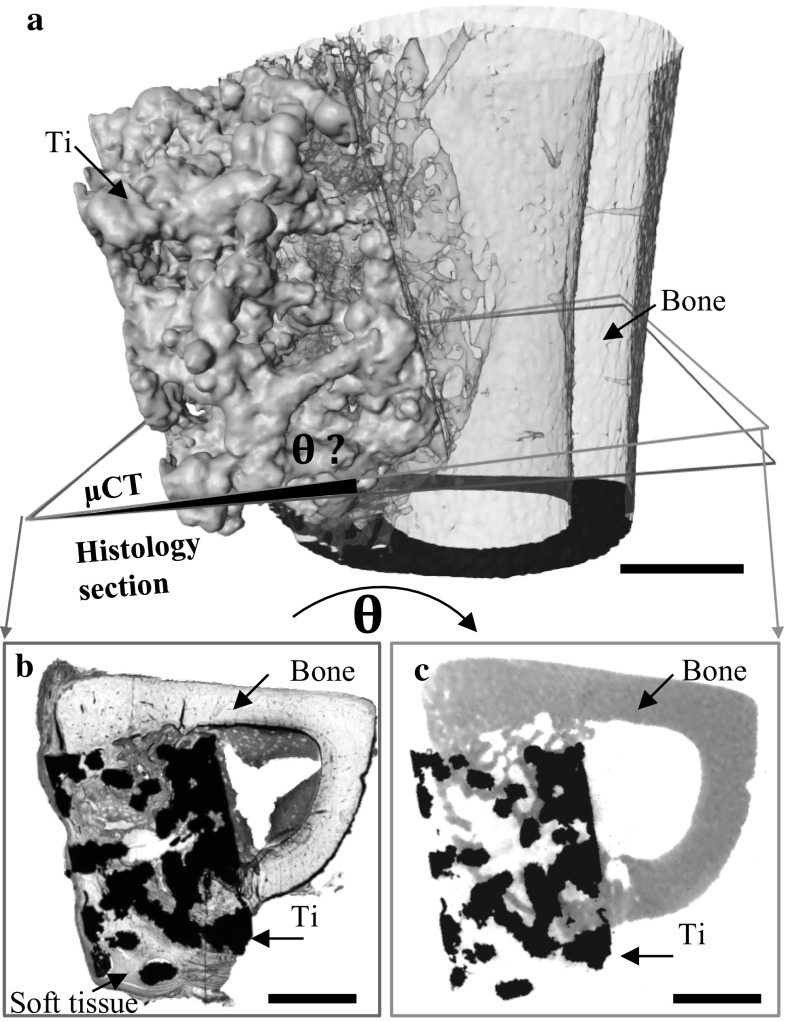
3D rendering of a 3 mm titanium scaffold within a rat tibiae defect illustrating a mismatch between the histology slice (*blue*) and the 2D slices of the μCT data (*green*). A multi-step registration was performed and final alignment was achieved between histology (**b**) and its corresponding 2D μCT images (**c**). *θ?* Indicates the unknown displacements and rotations required to register. *Scale bar* in all figures is 1000 μm (Color figure online)

The registration framework consisted of a segmentation stage for the porous titanium implant followed by two registration steps, as illustrated in Fig. 1. The segmented titanium scaffolds were used in the rigid registration to define the correspondence between the multimodal images independent of the surrounding bone and soft tissues. A more detailed description of the three steps of the registration is presented below.

Step 1 Segmentation of the titanium scaffold from the histological image and μCT volume.

The haematoxylin and multiple staining solution stained histology RGB (red, green and blue) images were split into the three RGB channels and the segmentation was performed on the red channel due to its superior brightness and contrast, which enabled easier segmentation of the histological image. An anisotropic diffusion filter was applied first to smooth the image while preserving the edge [29]. After that, global thresholding was applied to segment the titanium from the bone, allowing the modes of the histogram to be clearly distinguished in the 8-bit grayscale image. The thresholding value was acquired from the histogram by taking an average of the peak value of the titanium from three specimens.

Step 2 Coarse registration between the slice and volume datasets.

A search tool based on the correlation coefficient was used to identify the coarse correspondence between titanium segmented from histology and μCT (Fig. 1). The similarity between the porous titanium scaffold segmented from the μCT and histological results was defined by the correlation coefficient [30–32], implemented in Avizo (Visualisation Sciences Group, Merignac Cedex, France) and calculated as follows:

$$C = \frac{{\mathop \sum \nolimits_{i = 1}^{n} \left( { T_{i} - T_{m} } \right)*\left( { R_{i} - R_{m} } \right)}}{{\sqrt {\mathop \sum \nolimits_{i = 1}^{n} \left( {T_{i} - T_{m} } \right)^{2} } *\sqrt {\mathop \sum \nolimits_{i = 1}^{n} \left( {R_{i} - R_{m} } \right)^{2} } }}$$

where *T*_*i*_ is the intensity of the *i*^*th*^ pixel in the target image and *R*_*i*_ is the *i*^*th*^ intensity of the *i*^*th*^ pixel in the reference image. *T*_*m*_ and *R*_*m*_ are the mean intensity of the target image and reference image, respectively. A *C* value of 1 implies a positively identical registration between the two images.

Step 3 Refined registration of the target image.

Prior to the analysis, a parametric study was conducted to investigate the effect of the histological section thickness on the registration. If the thickness was equal to single voxel size, only a 2D transformation was allowed during the registration. Above 5 pixels, 6 degrees of freedom were allowed and the degree of similarity was stable. All the transformation metrics were recorded before validation.

### Accuracy of the registration method

A cylindrical titanium implant was scanned twice in conditions similar to those used for the implanted tibia. The porous implant was then cut using a diamond saw at 80^o^ to the axis of the cylinder.

The section was scanned at three different resolutions (2.5 μm/voxel, 4.5 μm/voxel, 9.0 μm/voxel). All data were processed and segmented based on the same protocol (Sect. 2.4).

The accuracy of the registration method was assessed by measuring the effect of image noise, scanning resolution and cutting (distortion) on the degree of similarity. To quantify the effect of image noise, a 2D μCT section was numerically extracted from the repeat scan and registered to the first scanned volume (reference). The cut section was then scanned at a different scanning resolution and registered to the reference volume. Finally, the CT sections before/after cutting were inspected visually to investigate any potential damage (material loss) and registered to assess any cutting induced distortion.

### Histological and 2D μCT quantification of bone formation

Once the transformation metrics were applied to the 3D μCT data, the histology correspondence 2D μCT could then be acquired. In addition, a defect equivalent region of interest (ROI) was acquired by applying the same method (in 2.4) to the transformed average leg model and cylinder mask.

Bone ingrowth and BC inside the 2D ROI and volumetric shrinkage were calculated for each histological image and its corresponding 2D μCT. During the histomorphometric analysis, each specimen was quantified over five random orientations using ImageJ [33] (*XY* grid). Values of BI and BC were calculated as an average of the five values for each specimen. All the measurements were performed for three specimens per time point and the results were based on the average of these measurements. The histology corresponding to the 2D μCT image was analysed using the same procedure as the 3D μCT.

The following parameters were investigated histologically and by 2D μCT: (1) BI: bone ingrowth [(bone area/ROI) × 100 %]; (2) BC: bone contact [(BC area/(total scaffold area) × 100 %]; (3) Volumetric shrinkage of bone: (a) [(cortical bone area histology/cortical bone area in 2D μCT) × 100 %] (b) [scale factor (*X*-axis) × scale factor (*Y*-axis)].

### Statistical analysis

All data were presented as a mean ± standard deviation. The Mann–Whitney U test was used for comparison of 3D μCT and 2D μCT/histological analysis across time points. A *p* value of < 0.05 was considered significant. After that, corresponding BI/BC values were used to show the regression line between histological and 2D μCT results. A 95 % confidence band for the regression line was applied (XLSTAT, 2014, Addinsoft, Inc., Brooklyn, NY, USA).

## Results

### Accuracy of registration

Satisfactory correlation was achieved after 39 fine iterations corresponding to a correlation ratio (C) of 0.85 (Supplementary Fig. 1) where a correlation ratio of 1 corresponds to a perfect match between the histology section and µCT data. Error in registration, i.e., lower correlation coefficient could result from noise in µCT data, difference in resolution of the two type of images and from sample deformation on histology section preparation. Among these factors, the sample deformation was found to be the major source of the mismatch (data not shown).

### Bone ingrowth (BI)

**Fig. 2:**
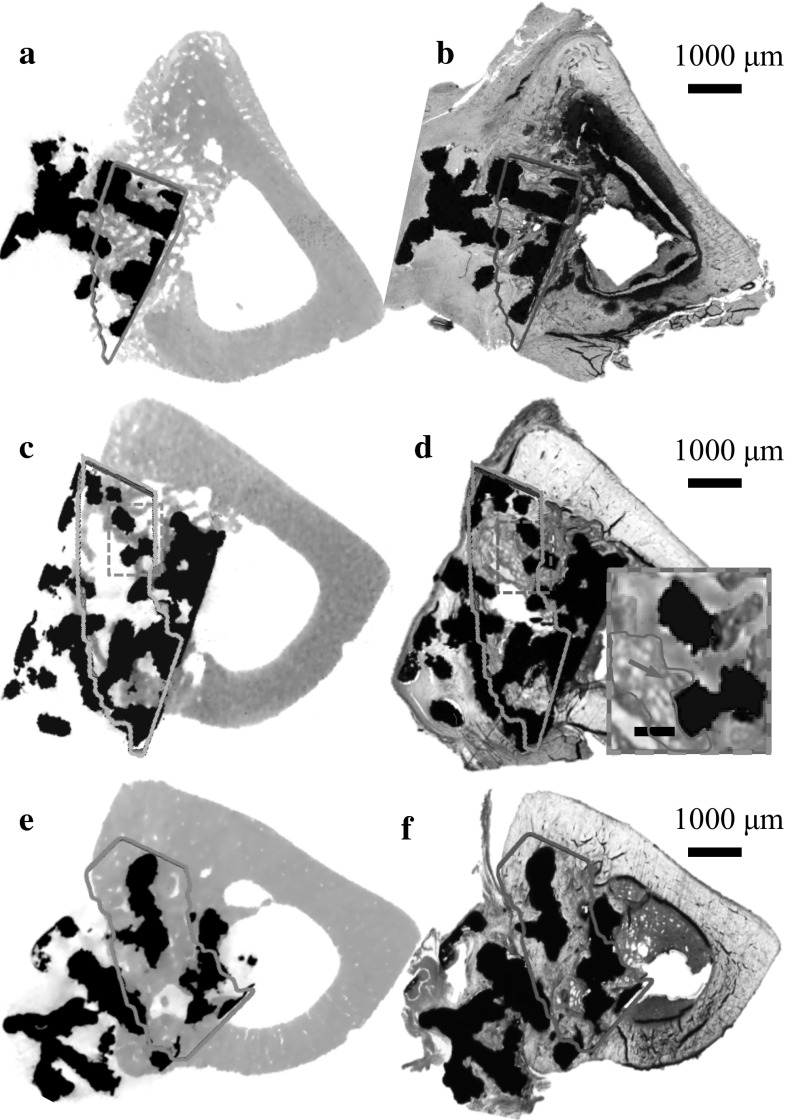
Examples of bone growth into the defect area at 2 (**a**, **b**), 3 (**c**, **d**) and 6 (**e**, **f**) weeks post-surgery. Bone formation and contact area were quantified in haematoxylin and multiple staining solution stained histological slices (ROI marked by *blue solid line*), which correlated well with its corresponding 2D μCT (ROI marked by *green solid line*). *Inset* of **d** shows zoomed in areas highlighted (*dashed line*) in **c** and **d** overlaid to show region of bone not detected (*blue arrow*) on μCT (Color figure online)

**Fig. 3:**
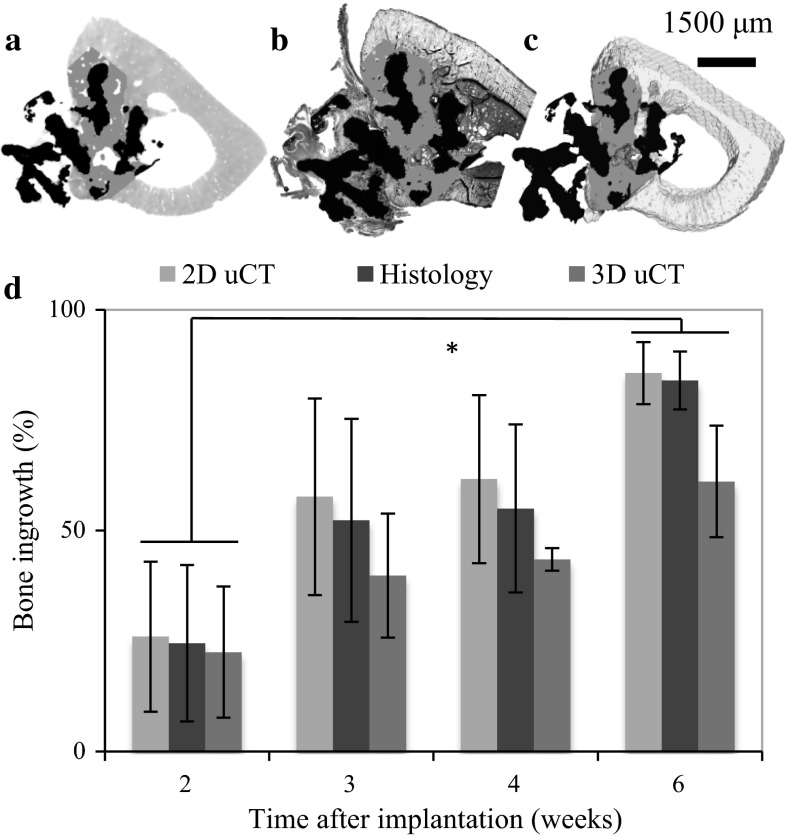
Bone ingrowth in corresponding **a** 2D μCT **b** histology and **c** 3D μCT image were measured as a function of time for 2, 3, 4 and 6 weeks (**d**). The quantity of newly formed bone increases significantly between 2 and 3 weeks and then after 4 weeks post-implantation

In total, twelve samples (n = 3 at 2, 3, 4 and 6 weeks’ post-implantation) were imaged and reconstructed. Figure 2 shows the corresponding 2D μCT slice and histology section for 2, 4 and 6 weeks following the semiautomated registration method. The defect equivalent ROI has good consistency between different specimens and time points. Results of quantitative data measured from 2D µCT/histology and 3D µCT have been summarised in Table 1 and plotted in Fig. 3. The BI increased with time as measured from both μCT data and histology images. Both 2D μCT and histology had similar BI for all time points. In comparison, the mean of BI measured from 3D μCT was lower than that of histology for all the time points, this was statistically significant at 6 weeks.

**Table 1:** Bone ingrowth into the porous titanium scaffold as a function of time measured from 2D μCT, histology and 3D μCT

Time (weeks)	BI (%)			BC (%)		
	2D µCT	Histology	3D µCT	2D µCT	Histology	3D µCT
2	25 ± 18	26 ± 17	23 ± 15	6 ± 9	7 ± 10	19 ± 14
3	52 ± 23	58 ± 22	40 ± 10	35 ± 27	26 ± 21	39 ± 19
4	55 ± 19	62 ± 19	44 ± 4	48 ± 21	48 ± 17	47 ± 2
6	84 ± 7	86 ± 7	61 ± 13	67 ± 11	72 ± 6	65 ± 9

**Fig. 4:**
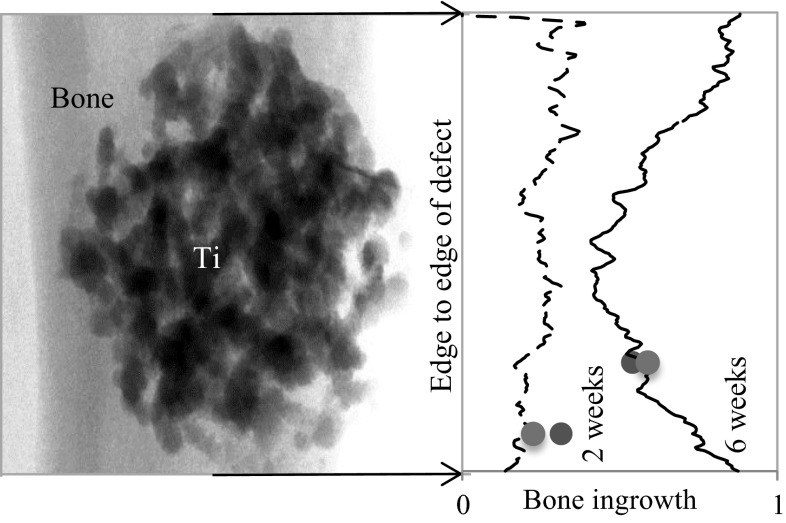
Bone ingrowth measured from 2D μCT slices and from edge to edge of a defect after 2 (*dashed line*) and 6 (*solid line*) weeks post-implantation. *Blue* and *green* *dots* on the *lines* mark the amount of bone ingrowth and location of histology sections and 2D μCT slices, respectively (Color figure online)

Figure 4 shows a plot of the bone formation within titanium implants from one end of the defect to the other after 2 and 6 weeks implantation. The exemplary plot demonstrates the increase in the amount of BI from 2 to 6 weeks. Further, after 6 weeks implantation the bone formation at the periphery was higher than at the core of the implant.

### Bone implant contact (BC)

**Fig. 5:**
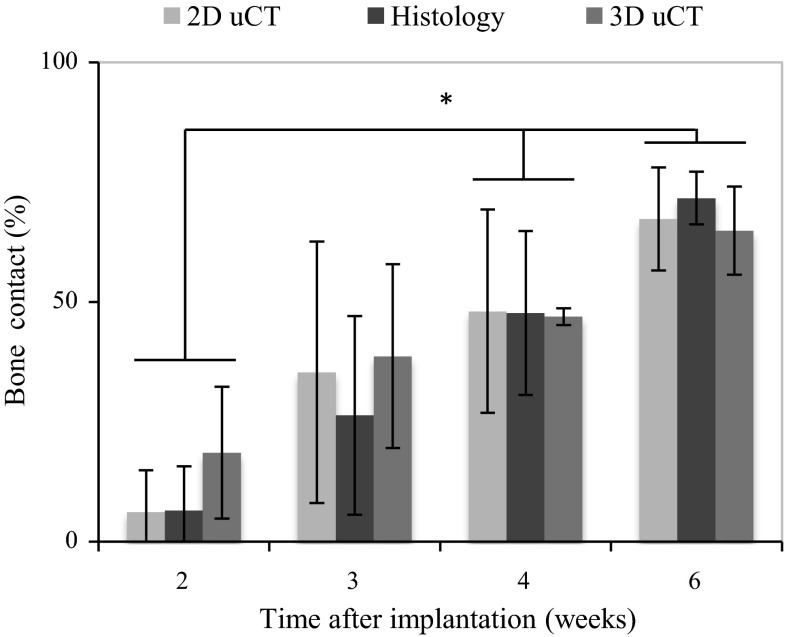
Bone implant contact was measured by 3D μCT, histology and its corresponding 2D μCT image. The amount of the bone implant contact shows a significant increase between 2 and 4 weeks, 2 and 6 weeks post-implantation

**Fig. 6:**
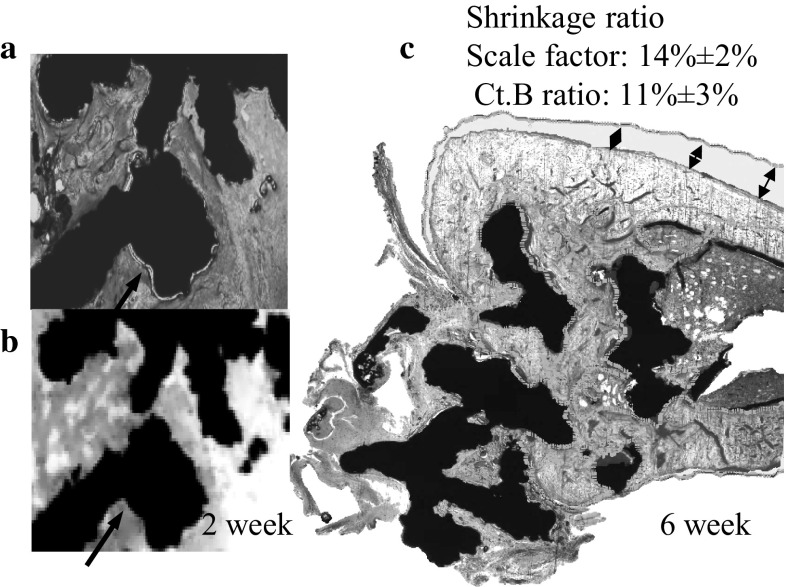
Tissue separation was observed on histology **a** in comparison to its corresponding 2D µCT slice **b** at 2 weeks post-implantation. Direct comparison of bone segmented from histology slices (haematoxylin and multiple staining solution stained tibiae) and its corresponding 2D µCT (marked as *green*) reveal a shrinkage (*black arrow*) of the bone tissue. Volumetric shrinkage was calculated based on scaling factor and cortical bone area ratio (**c**) (Color figure online)

Registration of histology sections to μCT data allows measurement of BC from the same region and comparison of data obtained via each method. Quantification of BC measured on histology sections, 2D μCT slices and 3D μCT data for the samples at different time points are shown in Table 1 and Fig. 5. All three measurements show that the BC increases with time where more than 65 % of the titanium surface had been covered with the newly formed bone after 6 weeks of implantation. Figure 6 shows registered images of histology sections and 2D μCT slice after 2 and 6 weeks implantation. Tissue separation was observed at 2 weeks implantation while extensive tissue shrinkage (~15 %) was after 6 weeks implantation.

## Discussion

Both μCT and histology methods have inherent advantages and limitations: with μCT a high number of slices can be generated per sample leading to volumetric information. A drawback is the relatively low resolution compared with histology. However, using a combination of μCT and histology, it is possible to perform a quantitative study in the same region. By using the non-deformed titanium (Supplementary Fig. 2) as the feature that was common in both μCT data and histology images registration was successfully performed. Importantly the accuracy of the registration was not affected by staining artefacts or soft tissue deformation. Figure 2 shows the corresponding 2D μCT slice and histology section for 2, 4 and 6 weeks following the semiautomated registration method.

### Bone ingrowth (BI)

The BI increased with time as measured from both μCT data and histology images. In the case of 2D μCT and histology, no significant differences in BI were observed at any of the time points. The data obtained showed a high correlation (*r* = 0.99) of BI between 2D μCT and histology, suggesting that that the semiautomated registration process adopted in this study is robust and 2D μCT has promise for achieving comparable results as histology in terms of quantifying BI. However, a difference in BI (mean) between 3D μCT and histology analysis was revealed at 2, 3, 4 and 6 weeks (Table 1; Fig. 3) this was significant at 4 and 6 weeks. This is due to a combination of preferential BI in porous implants and histological sectioning method that has a bias towards the edge of the implant hence the BI measured from histology sections is always an overestimation of the average BI in the whole implant. Figure 4 shows a representative plot of the BI from edge to edge of the implants after 2 and 6 weeks post-implantation measured from 2D μCT slices. It shows that BI is consistent throughout the implant 2 weeks after implantation. After 6 weeks, significant increase in bone growth is observed; however, the edge to edge profile (Fig. 4) of BI is different to that after 2 weeks. At 6 weeks, preferential BI is observed where, at the edges >70 % of the pores are filled with new bone while at the core only ~50 % of the void space had bone. This fits well with the observation by Muschler et al. [34]. who show that once an osteoconductive porous implant is fitted into a defect, the porous structure provides channels for cell attachment and migration. The newly formed bone grows in from the edge, continuing to grow towards the core of the implant while the bone at the edge remodels. In the case of 2 weeks, the low attenuation coefficient of the non-mineralised bone at the early time points (2 and 3 weeks) cannot be resolved on μCT (Fig. 2d inset), this may explain the flat profile seen on Fig. 4. However, after 6 weeks’ implantation, the majority of the bone is remodelled to a mineralised form which could be detected with confidence using μCT. This result suggests that 3D μCT is complimentary to histomorphometry analysis where the BI measured using 3D μCT is an average of BI of the whole implant, both edge and centre, to give a more representative result for bone formation, which is inherently volumetric.

### Bone contact (BC)

In this study, the mean of BC-histology is smaller than that of 3D μCT at 2 and 3 weeks. This is because the BC is more sensitive to the deformation of the newly formed bone within the implant surface on sample preparation for histology [35]. This situation changes at weeks 4 and 6, where, the BC-histology is larger than that of 3D μCT, suggesting that the bone had remodelled and adhered well to the implant. Figure 6 shows examples of tissue deformation on histology section preparation, in particular after 2 weeks (Fig. 6a); the tissue adjacent to the implant separated away from the surface of the implant leading to a lower measure of the BC from histology images (Table 1). Figure 6b shows that the volumetric shrinkage of cortical bone through histology section preparation 6 weeks after implantation could be as high as 15 %. Although, there was a large amount of tissue shrinkage and deformation, the tissue separation from the implant was negligible. This could be due to large amount of mature bone occupying majority of the void space that gives rise to stable bone-implant interface, which enables calculation of BC with confidence from histology sections. However, Fig. 6 undoubtedly reaffirms the need for a non-destructive means of measuring BC.

## Conclusions

A methodology was developed to perform semi-automatic correlative imaging using both μCT and quantitative histology (histomorphometry), allowing quantitative analysis of BI into titanium additive manufactured lattice implants. The evolution of bone density and quality over a 6 week period was tracked using BI and BC. It was shown that μCT provides full volumetric information, a higher degree of statistical significance, and is a simple and fast experimental technique. Histomorphometry is shown to complement μCT, providing higher resolution local information, particularly at early stages of BI.

The benefits of using correlative μCT and histomorphometric assessment over the gold standard technique of histology alone were demonstrated. Two potential areas where bias can be introduced when using histology alone are identified: errors due to the histology section measuring only a single plane within an complex 3D implant; and shrinkage during preparation of the histology section. The latter volumetric shrinkage error was quantified, and for the histology preparation used was ~15 %.

The correlative imaging methodology was then applied to quantify BI into an additive manufactured titanium lattice implant, quantifying the excellent BI and attachment achieved.

## Electronic supplementary material

Below is the link to the electronic supplementary material.

Supplementary Fig. 1 Correlation coefficient gradually converges to a large positive value (0.8–0.9), suggesting the registration process has been completed (TIF 579 kb)

Supplementary Fig. 2 SEM micrographs of the porous titanium scaffold prior to implantation, **a** scaffold top view **b** scaffold side view and **c** visualisation of scaffold strut thickness showing the porosity and roughness acquired (TIF 1250 kb)
