# Supplementary figures and images for: A correlative imaging based methodology for accurate quantitative assessment of bone formation in additive manufactured implants
Source: J Mater Sci Mater Med. 2016 May 6;27:112. doi: 10.1007/s10856-016-5721-6 (PMC4859838; doi:10.1007/s10856-016-5721-6)

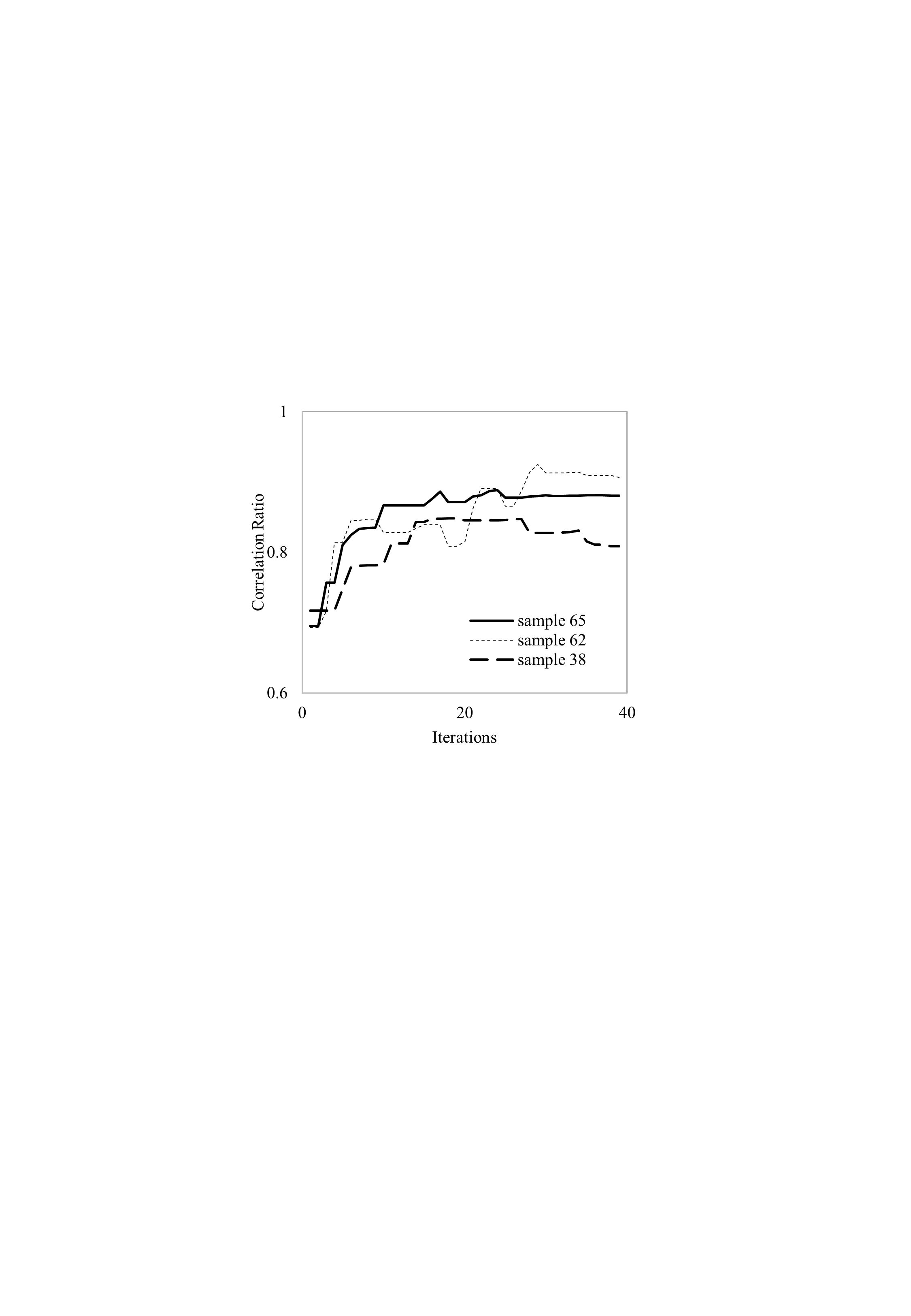

Supplement: Supplementary file 1 — Correlation coefficient gradually converges to a large positive value (0.8–0.9), suggesting the registration process has been completed (TIF 579 kb) [file 10856_2016_5721_MOESM1_ESM.tif]

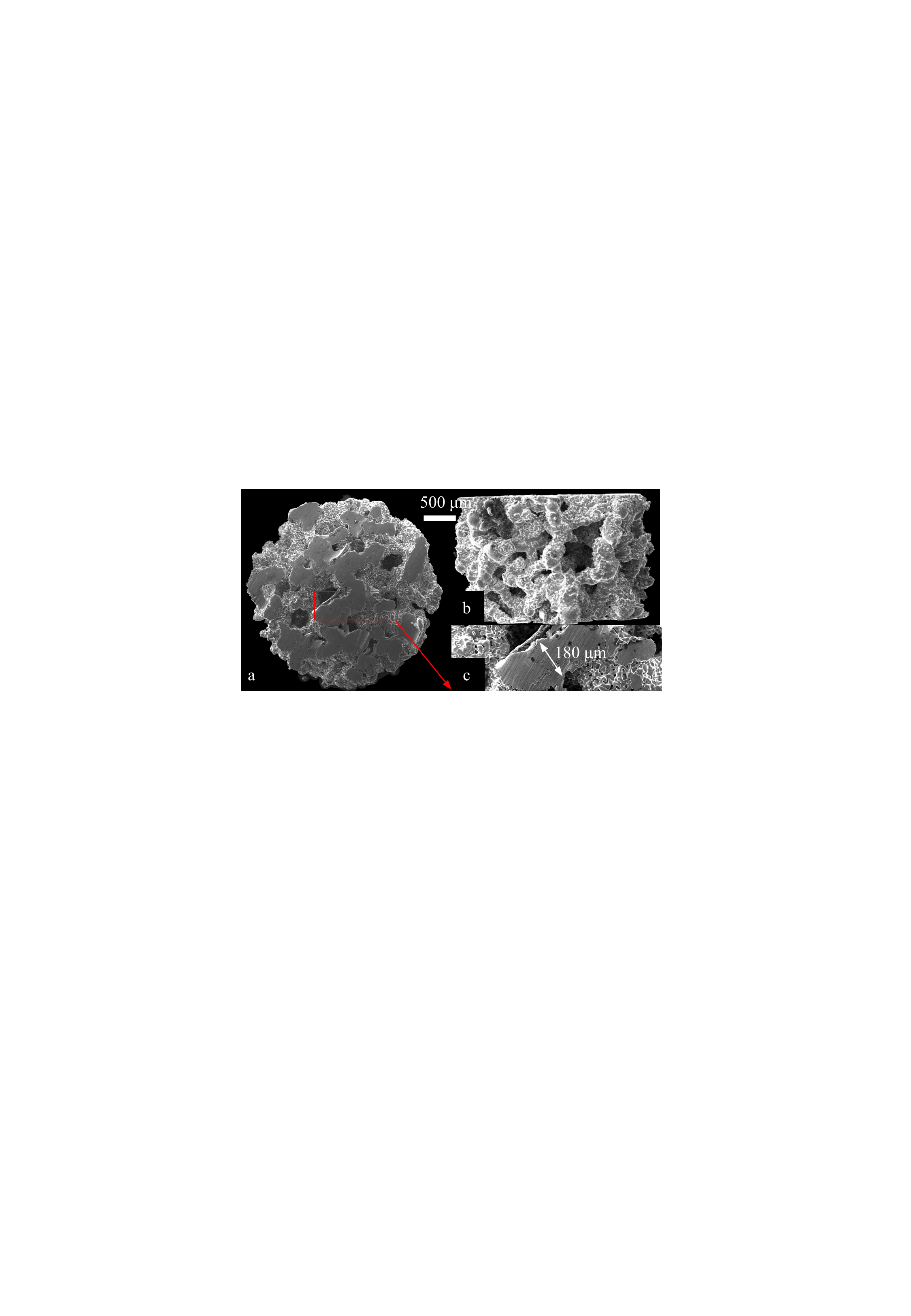

Supplement: Supplementary file 2 — SEM micrographs of the porous titanium scaffold prior to implantation, a scaffold top view b scaffold side view and c visualisation of scaffold strut thickness showing the porosity and roughness acquired (TIF 1250 kb) [file 10856_2016_5721_MOESM2_ESM.tif]
